# Calling for improved quality in the registration of traditional Chinese medicine during the public health emergency: a survey of trial registries for COVID-19, H1N1, and SARS

**DOI:** 10.1186/s13063-021-05113-y

**Published:** 2021-03-05

**Authors:** Zhuoran Kuang, Xiaoyan Li, Jianxiong Cai, Yaolong Chen, Xiaoyuan Qiu, Xiaojia Ni, on behalf of the Evidence-based Traditional and Integrative Medicine Working Group for Public Health Emergency

**Affiliations:** 1 Guangdong Provincial Hospital of Chinese Medicine, Guangdong Provincial Academy of Chinese Medical Sciences, The Second Clinical School of Guangzhou University of Chinese Medicine, Guangzhou, 510120 China; 2 Evidence-based Medicine Centre, School of Basic Medical Sciences, Lanzhou University, Lanzhou, 730000 China; 3 Chinese GRADE Centre, Lanzhou University, Lanzhou, 730000 China; 4 WHO Collaborating Centre for Guideline Implementation and Knowledge Translation, Lanzhou, 730000 China; 5 Guangzhou University of Chinese Medicine, Guangzhou, 510720 China; 6 Guangdong Provincial Key Laboratory of Research on Emergency in TCM, Guangzhou, 510120 China

**Keywords:** Traditional Chinese medicine, Clinical trial registration, WHO Trial Registration Data Set, COVID-19, H1N1, SARS

## Abstract

**Objective:**

To assess the registration quality of traditional Chinese medicine (TCM) clinical trials for COVID-19, H1N1, and SARS.

**Method:**

We searched for clinical trial registrations of TCM in the WHO International Clinical Trials Registry Platform (ICTRP) and Chinese Clinical Trial Registry (ChiCTR) on April 30, 2020. The registration quality assessment is based on the WHO Trial Registration Data Set (Version 1.3.1) and extra items for TCM information, including TCM background, theoretical origin, specific diagnosis criteria, description of intervention, and outcomes.

**Results:**

A total of 136 records were examined, including 129 severe acute respiratory syndrome coronavirus 2 (COVID-19) and 7 H1N1 influenza (H1N1) patients. The deficiencies in the registration of TCM clinical trials (CTs) mainly focus on a low percentage reporting detailed information about interventions (46.6%), primary outcome(s) (37.7%), and key secondary outcome(s) (18.4%) and a lack of summary result (0%). For the TCM items, none of the clinical trial registrations reported the TCM background and rationale; only 6.6% provided the TCM diagnosis criteria or a description of the TCM intervention; and 27.9% provided TCM outcome(s).

**Conclusion:**

Overall, although the number of registrations of TCM CTs increased, the registration quality was low. The registration quality of TCM CTs should be improved by more detailed reporting of interventions and outcomes, TCM-specific information, and sharing of the result data.

**Supplementary Information:**

The online version contains supplementary material available at 10.1186/s13063-021-05113-y.

## Introduction

Clinical trial registration is the process of registering critical information on medical research in publicly accessible databases to allow transparency and facilitate the design and implementation of a myriad of clinical trials [1, 2]. In 2004, a plan for implementing a global registry of clinical trials under the name of the International Clinical Trial Registration Platform (ICTRP) by the World Health Organization (WHO) emerged. In China, one of the primary registers of ICTRP, the Chinese Clinical Trial Registry (ChiCTR), was established in 2006 and given responsibility for submitting registered records to ICTRP [3]. Recent years have witnessed the following progress in the registration of clinical trials in China. First, prior to ethics committee approval, trials could register to obtain the assignment of a unique identification number. Second, registration in the Chinese Clinical Trial Registry is a necessary component in the application to conduct medical research projects. Furthermore, as of March 14, 2016, the ChiCTR has required registrants to include information on individual participant data (IPD) management and sharing plans [4].

Public health emergencies are major epidemics of infectious diseases, mass unexplained diseases, major food and occupational poisoning, and other events that seriously affect public health [5], such as severe acute respiratory syndrome coronavirus 2 (COVID-19), H1N1 influenza (H1N1), and severe acute respiratory syndrome (SARS), which have all caused large-scale epidemics in China. Since the safety and efficacy of any intervention in a public health emergency are unknown at the outset, it is particularly important in public health emergencies to register clinical trials before they are conducted to protect the safety of participants, ensure the scientific nature of the protocols implemented, and facilitate transparent supervision of the overall process of research. Traditional and complementary medicine (T&CM) is used in various countries for clinical practice and has been used to combat public health emergencies in some countries, such as Mali [6]. In China, national and regional policies and regulations have been implemented to guide the safe and effective use of T&CM. Therefore, countries where T&CM is widely used need to particularly focus on the quality of T&CM clinical trial registration.

Few studies have focused on the registration quality of traditional Chinese medicine clinical trials (TCM CTs) for H1N1, though some have touched upon TCM CTs of COVID-19 from the perspective of bibliometrics [7]. However, research assessing the registration quality specific to the TCM CTs of both remained untouched. Thus, we conducted this study to evaluate the registration quality specific to TCM CTs, which is of paramount significance during the current COVID-19 crisis. We took COVID-19, H1N1, and SARS as examples of public health emergencies because they were epidemics in China for which TCM was widely used. We aim to assess the registration quality of TCM CTs, which reflects their compliance with the WHO minimal data set [8], and by comparing the registration quality of TCM CTs recorded for COVID-19, H1N1, and SARS, we hope to explore the progress, if any, of TCM CTs in China.

## Methods

### Inclusion and exclusion criteria

#### Inclusion criteria

##### Types of diseases

All records of clinical trials that investigated COVID-19, H1N1, and SARS were included. The SARS outbreak occurred before the endorsement of trial registration, but trial registration was promoted immediately after, and retrospective registration was possible. We choose to include SARS to track quality from the beginning of trial registration. COVID-19 is defined by diagnostic criteria including “Diagnosis and treatment scheme for 2019-nCoV (Trial Version 1-7)” and WHO guidelines for the novel coronavirus. H1N1 is defined by diagnostic criteria including “Diagnosis and the treatment scheme of influenza” in China and WHO guidelines for the diagnosis of swine flu. SARS is defined by diagnostic criteria including the diagnosis and treatment scheme for SARS and WHO guidelines for the diagnosis of SARS. The diagnostic criteria were supported by laboratory or hypothetical evidence.

##### Types of intervention

All records of clinical trials that included intervention by traditional Chinese medicine or integrated traditional Chinese and Western medicine were included. TCM interventions included Chinese herbal formulas (decoctions, pills, powders, granules, ointments, etc.), Chinese herbal products (pills, tablets, pods, capsules, etc.), the injection of Chinese medicinal extracts, acupuncture (electric acupuncture, ear acupuncture, acupoint therapy, etc.), moxibustion, tuina (massage), cupping, guasha (scraping), Qigong, Tai Chi, and Ba Duan Jin. The dosage and route of administration were not restricted.

##### Types of study

Registered records of clinical trials that pertained to cross-sectional studies, cohort studies, non-randomized controlled trials, and randomized controlled trials were included.

No restriction was placed upon the language of the publication, trial participants, or recruitment.

#### Exclusion criteria

Records of duplicate trials (with the same trial ID) were excluded.

### Retrieval strategy

The WHO ICTRP database (https://www.who.int/ictrp/en/), which collects trial records from all trial registries worldwide such as ClinicalTrials.gov [9] and ChiCTR (http://www.chictr.org.cn/about.aspx), was searched on March 2, 2020, for information on relevant trials. The update search was conducted up to April 30, 2020. For more details on the search strategy, see Supplementary material 1.

### Registration quality assessment

The assessment of TCM CT registration quality was conducted based on WHO Trial Registration Data Set (TRDS) with a total of 24 items in “International Standards for Clinical Trial Registries” version 3.0 [10]. For better evaluation, the 24 items were divided into two parts: (1) common items (i.e., items 1–12, 16–18, 22, and 24) and (2) special items (i.e., items 13–15, 19–21, and 23), which contained multiple subitems that needed to be evaluated individually.

To assess the registration quality specific to TCM CTs, information on TCM theory, treatment principles, formulas, and herbs was examined. With reference to the SPIRIT-TCM Extension and the CONSORT Extension for Chinese Herbal Medicine Formulas 2017 [11, 12] and previous studies [13, 14], we included 4 additional items (hereafter referred to as TCM items) for assessing this quality. (1) TCM theoretical foundation, (2) TCM diagnostic criteria, (3) TCM intervention, and (4) TCM outcome were included for assessment.

A binary outcome variable was used to denote an item as “YES” for a complete report or “NO” for an incomplete or absent report based on the following denotations.

1. The TCM theoretical foundation reflects what (name a theory) a treatment is based on and where (name a classic) it comes from.
2. TCM diagnostic criteria are grounded on the identification of disease patterns (Zheng), which is a summary of the cause, nature, and location of pathologic changes at a certain stage of a disease.
3. TCM intervention varies from Chinese herbal formulas, Chinese herbal products, and acupuncture to other nonpharmaceutical treatments.
4. TCM outcome reveals the effectiveness of the TCM treatment, such as on a scale of TCM disease patterns.

### Data extraction and analysis

Two researchers (P Zeng and ZR Kuang) extracted data independently from all included records. Disagreement was resolved through discussion with the third researcher (XJ Ni). Data consist of two parts: (1) the minimum 24 items and of the WHO ICTRP Trial Registration Data Set (TRDS) and (2) 4 additional items for trials denoted as TCM CTs.

Data were imported into Microsoft Excel 2016 for descriptive statistical analysis of the baseline information, including (1) distribution of years and registries; (2) category of sponsor institution, study type, and intervention type; (3) characteristics of trial design and recruitment status; (4) prospective and retrospective registration; and (5) date for public access and reporting rate quality assessment items. The resulting data are presented as numbers (*n*) and percentages (%).

## Results

**Fig. 1:**
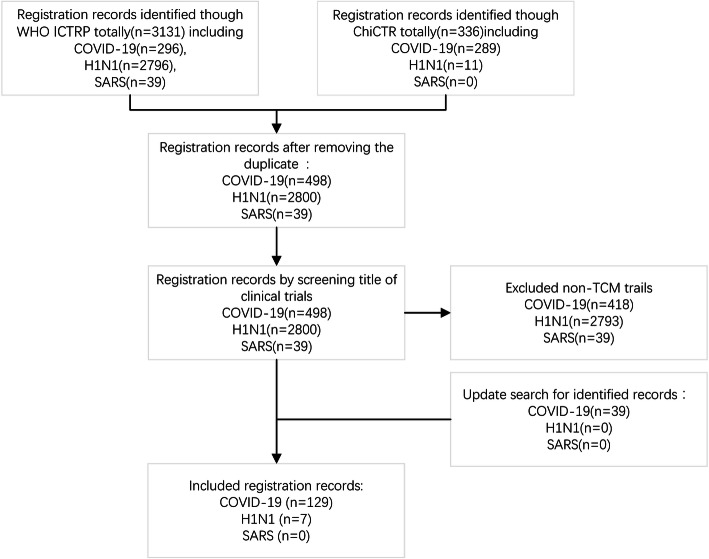
Flow diagram of record screening. ICTRP, International Clinical Trials Registry Platform; ChiCTR, Chinese Clinical Trial Registry; TCM, traditional Chinese medicine; COVID-19, coronavirus disease 2019; H1N1, H1N1 influenza; SARS, severe acute respiratory syndrome

The initial search identified 300 records from ChiCTR (including 289 COVID-19, 11 H1N1, 0 SARS) and 3131 records of ICTRP (including 296 COVID-19, 2796 H1N1, 39 SARS). Those that were not TCM CTs and duplicates were excluded. A total of 39 SARS-related CTs were excluded because of not involvement of TCM. After rigorous screening, our study identified 90 TCM CT entries for COVID-19 and 7 for H1N1. As an updated search on April 30 found 39 more records for COVID-19, our final study was able to cover 129 registered entries for COVID-19 and 7 registered entries for H1N1. For details, see Fig. 1.

### Basic characteristics of TCM CT registration

All 129 TCM CTs for COVID-19 were registered in 2020, while those for H1N1 were registered in 2009 and 2010. Registrations of COVID-19 were found in 2 registries: ChiCTR (94.6%, 122/129) and ClinicalTrials.gov (5.4%, 7/129), while those of H1N1 were found in 2 registries: ChiCTR (71.4%, 5/7) and ClinicalTrials.gov (28.6%, 2/7). And all the included TCM CTs were implemented in China.

The common primary sponsor of TCM CTs found in registries was a hospital: 82.9% (107/129) registered trials of COVID-19 and 71.4% (5/7) of H1N1. Interventional studies (82.5%, 112/136) held the leading position. There were a variety of TCM interventions, in which Chinese herbal products (33.8%, 46/136) contributed to the largest proportion, followed by Chinese herbal formulas (29.4%, 40/136). In addition, a proportion of records provided only ambiguous wording regarding “TCM treatment” (14.0%, 19/136) in this field.

The common design was randomized (64.7%, 88/136) and parallel (63.2%, 86/136) studies. Only a few trials applied blinding, including double blind (6.6%, 9/136), triple blind (0.7%, 1/136), and quadruple blind (0.7%, 1/136) trials. Most (64.7%, 88/136) did not report any information on blinding at all. The main recruitment status was recruiting (52.2%, 71/136) and pending (42.6%, 58/136).

Prospective registrations included all registered records for H1N1 and 85% (110/129) for COVID-19.

Most registrants provided details on the plan to publicly share IPD, including 92% (119/129) of COVID-19 TCM CTs and 86% (6/7) of H1N1 TCM CTs.

Summary of TCM CT records as per Table 1.

**Table 1:** Summary of TCM CT records (*n*, %)

Category	COVID-19 (*N* = 129)	H1N1 (*N* = 7)	Total (*N* = 136)
**Registration**			
ChiCTR	122 (94.6%)	5 (71.4%)	127 (93.4%)
ClinicalTrials.gov	7 (5.4%)	2 (28.6%)	9 (6.6%)
**Study type**			
Interventional study	107 (82.9%)	5 (71.4%)	112 (82.4%)
Observational study	22 (17.1%)	2 (28.6%)	24 (17.6%)
**Primary sponsor**			
Hospital	107 (82.9%)	5 (71.4%)	112 (82.4%)
University	12 (9.3%)	N/A	12 (8.8%)
Others	10 (7.8%)	2 (28.6%)	12 (8.8%)
**Intervention**			
Chinese herbal products	42 (32.6%)	4 (57.1%)	46 (33.8%)
TCM treatment	16 (12.4%)	3 (42.9%)	19 (14.0%)
Chinese herbal formula	40 (31.0%)	N/A	40 (29.4%)
Integration of traditional Chinese and Western medicine	11 (8.5%)	N/A	11 (8.1%)
Nonpharmaceutical treatment of TCM	8 (6.2%)	N/A	8 (5.9%)
Others	12 (9.3%)	N/A	12 (8.8%)
**Assignment**			
Parallel	81 (62.8%)	5 (71.4%)	86 (63.2%)
Sequential	15 (11.6%)	N/A	15 (11.0%)
Single arm	7 (5.4%)	N/A	7 (5.1%)
Factorial	2 (1.6%)	N/A	2 (1.5%)
Others	5 (3.9%)	2 (28.6%)	7 (5.1%)
Not reported	19 (14.7%)	N/A	19 (14.0%)
**Method of allocation**			
Randomized	83 (64.3%)	5 (71.4%)	88 (64.7%)
Non-randomized	45 (34.9%)	2 (28.6%)	47 (34.6%)
Quasi-randomized	1 (0.8%)	N/A	1 (0.7%)
**Masking**			
Quadruple blind	1 (0.7%)	N/A	1 (0.7%)
Triple blind	1 (0.7%)	N/A	1 (0.7%)
Double blind	8 (6.2%)	1 (14.3%)	9 (6.6%)
Open label	36 (27.9%)	1 (14.3%)	37 (27.2%)
Not reported	83 (64.3%)	5 (71.4%)	88 (64.7%)
**Recruitment status**			
Pending	58 (45.0%)	N/A	58 (42.6%)
Recruiting	68 (52.7%)	3 (42.9%)	71 (52.2%)
Suspended	2 (1.6%)	N/A	2 (1.5%)
Completed	1 (0.8%)	4 (57.1%)	5 (3.7%)
**Prospective and retrospective registration**			
Retrospective	19 (14.7%)	N/A	19 (14.0%)
Prospective	110 (85.3%)	7 (100.0%)	117 (86.0%)
**IPD sharing plan**			
Yes	119 (92.2%)	6 (85.7%)	125 (91.9%)
No	3 (2.3%)	N/A	3 (2.2%)
N/A	7 (5.4%)	1 (14.3%)	8 (5.9%)

*ChiCTR* Chinese Clinical Trial Registry, *TCM* traditional Chinese medicine, *COVID-19* severe acute respiratory syndrome coronavirus 2, *H1N1* H1N1 influenza, *N/A* not available

### Registration quality assessment

#### Common items

The reporting rate of 17 common items varied from 75.7 to 100% in all registered TCM CTs. Most (> 90%) covered the following items: source(s) of monetary or material support, secondary sponsor(s), contact for public queries, contact for scientific queries, completion date, and data sharing plan. In addition, 75.7% of TCM CTs recorded secondary identification numbers. The other common items were all presented in the entries of TCM CTs. For details, see Table 2.

**Table 2:** Quality assessment of registration information on common items (*n*, %)

No. Item name	COVID-19 (*n* = 129)	H1N1 (*n* = 7)	Total (*n* = 136)
1. Primary registry and trial identifying number	129 (100.0%)	7 (100.0%)	136 (100.0%)
2. Date of registration in primary registry	129 (100.0%)	7 (100.0%)	136 (100.0%)
3. Secondary identifying numbers	97 (75.2%)	6 (85.7%)	103 (75.7%)
4. Source(s) of monetary or material support	127 (98.4%)	7 (100.0%)	134 (98.5%)
5. Primary sponsor	129 (100.0%)	7 (100.0%)	136 (100.0%)
6. Secondary sponsor(s)	121 (93.8%)	5 (71.4%)	126 (92.6%)
7. Contact for public queries	128 (99.2%)	7 (100.0%)	135 (99.3%)
8. Contact for scientific queries	128 (99.2%)	7 (100.0%)	135 (99.3%)
9. Public title	129 (100.0%)	7 (100.0%)	136 (100.0%)
10. Scientific title	129 (100.0%)	7 (100.0%)	136 (100.0%)
11. Countries of recruitment	129 (100.0%)	7 (100.0%)	136 (100.0%)
12. Health condition(s) or problem(s) studied	129 (100.0%)	7 (100.0%)	136 (100.0%)
16. Date of first enrollment	129 (100.0%)	7 (100.0%)	136 (100.0%)
17. Target sample size	129 (100.0%)	7 (100.0%)	136 (100.0%)
18. Recruitment status	129 (100.0%)	7 (100.0%)	136 (100.0%)
22. Completion date	119 (92.2%)	6 (85.7%)	125 (91.9%)
24. Data sharing plan	122 (94.6%)	6 (85.7%)	128 (94.1%)
Total common items average report percentage (%)	97.2%	95.8%	97.1%

*COVID-19* severe acute respiratory syndrome coronavirus 2, *H1N1* H1N1 influenza

#### Special items

The total average reporting rate of the 7 special items presented was 45.6%. Most (82.4%) provided information on ethics reviews, but none of those provided summary results. For details, see Table 3.

**Table 3:** Quality assessment of registration information on special items (*n*, %)

No. Item name	COVID19 (*n* = 129)	H1N1 (*n* = 7)	Total (*n* = 136)
13. Interventions			
13.1 Intervention name	129 (100.0%)	7 (100.0%)	136 (100.0%)
13.2 Ingredients or detail technique	13 (10.1%)	1 (14.3%)	14 (10.3%)
13.3 Form	55 (42.6%)	4 (57.1%)	59 (43.4%)
13.4 Dosage and frequency	20 (15.5%)	4 (57.1%)	24 (17.6%)
13.5 Treatment duration	6 (4.7%)	0 (0.0%)	6 (4.4%)
13.6 Control group	103 (79.8%)	7 (100.0%)	110 (80.9%)
Total average	54.3 (42.1%)	3.8 (54.8%)	58.1 (42.7%)
14. Key inclusion and exclusion criteria			
14.1 Criteria of inclusion and exclusion	129 (100.0%)	7 (100.0%)	136 (100.0%)
14.2 Gender	129 (100.0%)	7 (100.0%)	136 (100.0%)
14.3 Age	109 (84.5%)	7 (100.0%)	116 (85.3%)
14.4 Diagnosis criteria—Western medicine	45 (34.9%)	2 (28.6%)	47 (34.6%)
14.5 With healthy human volunteer	1 (0.8%)	0 (0.0%)	1 (0.7%)
Total average	82.6 (64.0%)	4.6 (65.7%)	87.2 (64.1%)
15. Study type			
15.1 Type of study	129 (100.0%)	7 (100.0%)	136 (100.0%)
15.2 Method of allocation (randomized/non-randomized)	107 (82.9%)	5 (71.4%)	112 (82.4%)
15.3 Masking (is masking used and, if so, who is masked)	46 (35.7%)	2 (28.6%)	48 (35.3%)
15.4 Assignment (single arm, parallel, crossover, or factorial)	107 (82.9%)	7 (100.0%)	114 (83.8%)
15.5 Allocation concealment mechanism	6 (4.7%)	0 (0.0%)	6 (4.4%)
15.6 Phase (if applicable)	79 (61.2%)	5 (71.4%)	84 (61.8%)
Total average	79.0 (61.2%)	4.3 (61.9%)	83.3 (61.3%)
19. Primary outcome(s)			
19.1 Name	128 (99.2%)	7 (100.0%)	135 (99.3%)
19.2 Measurement of primary outcome(s)	2 (1.6%)	0 (0.0%)	2 (1.5%)
19.3 Time point of measurements	17 (13.2%)	1 (14.3%)	18 (13.2%)
Total average	49.0 (38.0%)	2.7 (38.1%)	51.7 (38.0%)
20. Key secondary outcome(s)			
20.1 Name	89 (69.0%)	7 (85.7%)	96 (70.6%)
20.2 Measurements of key secondary outcomes	2 (1.6%)	0 (0.0%)	2 (1.5%)
20.3 Time point of measurements	15 (11.6%)	1 (14.3%)	16 (11.8%)
Total average	35.3 (27.4%)	2.7 (38.1%)	38.0 (27.9%)
21. Ethics review			
21.1 Ethics review status	125 (96.9%)	6 (83.3%)	131 (96.3%)
21.2 Date of approval	98 (76.0%)	4 (69.0%)	102 (75.0%)
21.3 Name of ethics committee(s)	105 (81.4%)	4 (78.6%)	109 (80.1%)
21.4 Contact details of ethics committee(s)	104 (80.6%)	0 (40.5%)	104 (76.4%)
Total average	108.0 (83.7%)	4.0 (57.1%)	112.0 (82.4%)
23. Summary results			
23.1 Date of posting of result summaries	0 (0%)	0 (0%)	0 (0%)
23.2 Date of the first journal publication of results	0 (0%)	0 (0%)	0 (0%)
23.3 URL hyperlink(s) related to results or a full reference list of publications	0 (0%)	0 (0%)	0 (0%)
23.4 Baseline characteristics	0 (0%)	0 (0%)	0 (0%)
23.5 Participant flow, adverse events	0 (0%)	0 (0%)	0 (0%)
23.6 Adverse events	0 (0%)	0 (0%)	0 (0%)
23.7 Outcome measures	0 (0%)	0 (0%)	0 (0%)
23.8 URL link to protocol file(s) with version and date	0 (0%)	0 (0%)	0 (0%)
Changes to protocol	0 (0%)	0 (0%)	0 (0%)
Total average	0 (0%)	0 (0%)	0 (0%)
Total special items average report percentage (%)	45.6%	45.1%	45.6%

*COVID-19* severe acute respiratory syndrome coronavirus 2, *H1N1* H1N1 influenza

Less than half of the TCM CTs provided complete information on the intervention. They commonly reported the most basic information, including the name of the intervention (100.0%, 136/136) and control group (80.9%, 110/136). Information on the ingredients of Chinese herbal formulas and herbal products, selected acupoints of acupuncture, and other nonpharmaceutical treatments of TCM (Qigong, Tai Chi, and Ba Duan Jin), however, fell short. Information on the form, dosage, frequency, and duration of interventions was also inadequate. For example, only 6 (4.4%) TCM CTs provided information on treatment duration.

Most (> 80%) records showed information on the inclusion and exclusion criteria for gender and age. Only 34.6% (47/136) covered the diagnostic criteria based on Western medicine instead of TCM.

Study type was recorded in all TCM CTs. Most (> 80%) provided information on the method of allocation and assignment. A total of 61.8% (84/136) mentioned the trial phase, but only approximately one third, however, reported a masking method including whether it was used and, if so, who was masked. The least reported information (4.4%) on item 15 was the allocation concealment mechanism.

A total of 99.3% of TCM CTs registered primary outcome(s) and 70.6% recorded key secondary outcomes. The specifics of primary outcomes and key secondary outcomes, including measurement method and time point of measurements, however, were far from adequate and sometimes absent. For example, only 1.5% (2/136) mentioned how primary outcomes were measured, while 13.2% (18/136) provided the time point of measurements.

The average coverage of information on the ethics review was 82.4%, which included ethics review status (whether it was approved and, if so, date of approval), name of the ethics committee(s), and contact details of the ethics committee(s).

None of the eligible records provided follow-up information in the field of summary results such as date of submitting the results, URL hyperlink(s) to these results or a full reference list of publications, baseline characteristics, participant flow, adverse events, outcome measures, URL link to protocol file(s), or changes to the protocol, if any.

### Registration quality specific to TCM items

TCM CTs fell short in providing relevant information on our 4 additional TCM items, evidenced by the following reported percentages presenting in a descending order: on TCM outcome (27.2%, 37/136), on TCM intervention (5.9%, 8/136), and on TCM diagnostic criteria (5.9%, 8/136). Information on the TCM theoretical foundation was completely omitted (0.0%, 0/136). For details, see Table 4.

**Table 4:** Registration quality specific to TCM items (*n*, %)

Item name	COVID-19 (*n* = 129)	H1N1 (*n* = 7)	Total (*n* = 136)
Descriptions of TCM intervention	7 (5.4%)	1 (14.3%)	8 (5.9%)
TCM syndrome diagnosis	8 (6.2%)	0 (0.0%)	8 (5.9%)
TCM-specific outcomes	35 (27.1%)	2 (28.6%)	37 (27.2%)
TCM-specific background, rationale, theoretical origin	0 (0.0%)	0 (0.0%)	0 (0.0%)

*COVID-19* severe acute respiratory syndrome coronavirus 2, *H1N1* H1N1 influenza, *TCM* traditional Chinese medicine

## Discussion

The noninclusion of SARS-related registered clinical trials may be due to the registration of clinical trials that had not been endorsed when the SARS outbreak occurred. The outbreak of COVID-19 witnessed a higher growth rate in CTRs than the H1N1 crisis. Stagnant progress, however, was detected in the registration quality of clinical trials in the last decade. We also identified the same CT registration deficiencies during the H1N1 incident as found in the COVID-19 emergency, manifested in a lack of specifics on interventions and outcomes as well as the omission of follow-up results. More importantly, TCM CTs were found to have an incredibly low reporting percentage for TCM items.

Our study was the first to evaluate the overall registration quality specific to TCM CTs during public health emergencies. Compared to other similar studies, we assessed registration quality using the updated WHO TRDS version 1.3.1, which included four new items (i.e., ethics review, completion date, summary results, and IPD sharing statement) [15]. Our research compared the registration quality between the TCM CTs for H1N1 and those for COVID-19 and thus revealed the development of TCM CT registration. When comparing the registered TCM CTs of H1N1 and those of COVID-19, our findings suggest registration progress in following the routine policies of prospective registration and ethics reviews. These experiences and lessons could be applied to the clinical trial registration of other T&CMs, if any.

Data from previous studies found 39% of clinical trials identified as retrospective [13]; however, our study found that the registrations of TCM CTs conducted during two infectious disease outbreaks were almost all prospective (100% in H1N1 and 87% in COVID-19). Two reasons could account for the high compliance with prospective registration. First, the member journals of ICMJE reject retrospectively registered trials because these might be confined to their anticipated results [16]. Second, ChiCTR requires registrants to pay for data audit and database maintenance to prevent retrospective registration [17].

Compared with H1N1, more information on ethics reviews was reported in the TCM CTs of COVID-19, most of which (> 90%) provided details on the approval document of the ethics committee. This could be attributed to the fact that the National Medical Products Administration issued “Good Clinical Practice, GCP” in 2003 and “Guidelines for the Ethical Review of Drug Clinical Trials” in 2010, which specified the rights of the subjects and the working procedures of the ethics committee [18]. Ethics review is essential in all CTs, even during an acute disaster situation where standard procedures are modified to uphold ethical principles in the most expedient manner possible [19]. In such crises as COVID-19, when standard treatment is not available, approval by the ethics committee for investigational drugs is of paramount urgency, as it allows researchers to proceed with trials to look for effective cures without compromising oversight mechanisms. As a result, emergency meetings of the committee are more than necessary to ensure the rapid initiation of valuable studies under ethical protocols [20].

Our findings, on the other hand, suggest deficiencies in the registration of TCM CTs in reporting TCM items in the fields of interventions, outcomes, and result sharing plans over the last decade. In a country such as China, where TCM plays a significant role in its medical system equal to its role in research during public health emergencies, underreporting relevant information not only wastes medical and scientific resources but also undermines the scientific nature of TCM and the rationality of TCM research. Thus, registration quality specific to TCM CTs needs to be improved.

Inadequate information on interventions and outcomes is apparent in both cases of H1N1 and COVID-19. Registration improves the transparency of TCM CTs by making protocols available to the public. Publicity, however, is invalid if the results do not appear in a journal [21]. Therefore, it is necessary not only to provide sufficient information in the registration but also to provide a prerequisite for quality evaluation, i.e., to prevent reporting bias. When information on registration is incomplete, billions in investments are wasted and bias sets in, to the detriment of medical research and patient care [22].

Poor registration quality was accounted for by Viergever et al. as having two sources [23]. First, some data are mandatory to report while others optional. ChiCTR (almost all included CTs were registered in ChiCTR), for instance, requires registrants to provide methods of measurement and the time point of measurements for each outcome but does not enforce this. In addition, only a blank text field labeled “Description for medicine or protocol of treatment in detail” was available for entry for the intervention [17]. This could explain our finding in which some H1N1 and COVID-19 TCM CTs only provided the description “TCM treatment” (14.0%, 19/136) as the intervention. The wording “TCM treatment” is quite vague and lacks important details such as the ingredients of Chinese herbs, dosage, frequency, duration, and route of administration.

The second source is the inefficient quality control imposed by registries on registrants. ClinicalTrials.gov reviews whether CTRs are complete and meaningful [24], but ChiCTR lacks standard criteria in regulating registration quality. The registration guidelines of ChiCTR only stipulate that if the CTR is unclear, more information will be requested. With regard to quality control improvement, the mandatory submission of information such as specific measurement technique and time point(s) of outcome(s), as well as different tiers of data checking such as automated and manual checks, should be implemented to detect insufficient and nonmeaningful entries [23].

Our finding of insufficient reporting of TCM CTs on TCM items was supported by a recent study where the reporting percentage was found to be almost less than 50% [13]. Although the SPIRIT-TCM Extension and the CONSORT extension for Chinese herbal medicine formulas recommended integrated criteria for Western medicine and TCM in diagnosing disease and TCM outcome items to assess the effectiveness of interventions [11, 12], the reporting rate in question was low. This could be attributed to the lack of standard TCM CT items for data submission in the WHO registry [13]. Without transparent registered information on trial design and implementation, the results for TCM CTs can be questioned [7]. TCM CTs were conducted under the unique system of TCM theory, treatment principles, formulas, and herbs and thus are different from Western medical interventional CTs [11–13]. To date, several problems have been detected in the TCM CTs for COVID-19, including but not limited to the incorrect labeling of design type, ineligible inclusion and exclusion criteria, restricted feasibility of placebo comparator against Chinese herbs, and unclear information on the intervention and outcome [25]. These problems compromise not only the efficacy and safety of Chinese medicine but also reviewers’ and readers’ judgments on the value of TCM in general, inviting skepticism and criticism. Therefore, it is vital to provide complete and meaningful information on TCM items.

The lack of a plan to publicly share the IPD is another major problem encountered in our assessment of TCM CT registrations. The failure to support public access to full trial data compromises the authenticity of clinical trials [26]. The fact that clinical trials of COVID-19 are ongoing could be one important cause of this finding. Another is that ChiCTR allows registrants to share data a year after trial completion without special consideration for public emergencies [15]. Common accessible data, including contacts of the principal investigator, full study reports (detailing all analyses), journal reports, and participant-level datasets [22], are urgently needed, especially during an infectious disease outbreak, so that limited resources can be fully deployed to identify etiological factors, predict disease spread, evaluate existing and novel treatments, provide symptomatic care, and timely develop preventive measures [27]. Our study revealed, however, that data sharing was not taken seriously during the H1N1 crisis. This neglect was echoed by the 2013–2016 Ebola virus outbreak in West Africa. Fortunately, a protocol for data sharing and reporting named “COVID-19 Open” was implemented to improve timely access to data during the current COVID-19 emergency [28]. Despite the protocol call for a data sharing plan, none of our collected registrations of TCM CTs provided IPD summary results in ChiCTR but did in other databases, such as “COVID-19 Knowledge & Data Hub” [29]. China has shared information about COVID-19 to promote international cooperation in epidemic prevention and control [30].

In addition to the above issues, it is worth noting that most of the 129 TCM COVID-19 clinical trials we found would last approximately a year, and as the COVID-19 epidemic has been controlled in China, many studies may not be completed due to a lack of patients to include and will have to be terminated, which would be a great waste of resources. Therefore, in the registration of public health emergencies, the management of thematic registration should be strengthened or special teams should be set up to deal with the registration process, while the government should strengthen the coordination arrangements and rationalize the organization of clinical trials. In China, the New Coronary Pneumonia Outbreak Joint Prevention and Control Mechanism Research and Tackling Team was established to oversee clinical research on the COVID-19 epidemic [31].

Our study included several limitations. First, our last search was in early 2020, which might not cover all the TCM CTs for COVID-19. Second, WHO Trial Registration Data Set (TRDS) extension for traditional Chinese medicine 2020 has been published recently and it includes more items specific to TCM (Supplementary material 2) [32]. Future studies should update the search and use the TRDS-TCM for the registration quality assessment.

## Conclusion

From SARS to H1N1 and from H1N1 to COVID-19, an increase was detected in the number of registrations of TCM CTs. While progress was maintained in complying with policies on prospective registration and on ethical review, the overall registration quality of TCM CTs during infectious disease outbreaks remained poor. Future efforts are needed to improve the quality: (1) registrants of TCM CTs should report detailed information on interventions and outcome indicators, (2) registries should improve quality control on required information, and (3) guidelines for TCM CTRs should be implemented to oversee the completion of registration content.

## Supplementary Information

**Additional file 1.** Search strategy.

**Additional file 2.** Registration quality assessment of TCM CTs by using TRDS-TCM.

## Data Availability

The dataset supporting the conclusions of this article is included within the article.

## Abbreviations

ChiCTR: Chinese Clinical Trial Registry
COVID-19: Severe acute respiratory syndrome coronavirus 2
CT: Clinical trial
GCP: Good Clinical Practice
H1N1: H1N1 influenza
ICTRP: International Clinical Trial Registration Platform
IPD: Individual participant data
N/A: Not available
TCM: Traditional Chinese medicine
T&CM: Traditional and complementary medicine
WHO: World Health Organization
TRDS: Trial Registration Data Set
SARS: Severe acute respiratory syndrome
