# Correction to: Calling for improved quality in the registration of traditional Chinese medicine during the public health emergency: a survey of trial registries for COVID-19, H1N1, and SARS

**DOI:** 10.1186/s13063-021-05182-z

**Published:** 2021-04-07

**Authors:** Zhuoran Kuang, Xiaoyan Li, Jianxiong Cai, Yaolong Chen, Xiaoyuan Qiu, Xiaojia Ni, on behalf of the Evidence-based Traditional and Integrative Medicine Working Group for Public Health Emergency

**Affiliations:** 1 Guangdong Provincial Hospital of Chinese Medicine, Guangdong Provincial Academy of Chinese Medical Sciences, The Second Clinical School of Guangzhou University of Chinese Medicine, Guangzhou, 510120 China; 2 Evidence-based Medicine Centre, School of Basic Medical Sciences, Lanzhou University, Lanzhou, 730000 China; 3 Chinese GRADE Centre, Lanzhou University, Lanzhou, 730000 China; 4 WHO Collaborating Centre for Guideline Implementation and Knowledge Translation, Lanzhou, 730000 China; 5 Guangzhou University of Chinese Medicine, Guangzhou, 510720 China; 6 Guangdong Provincial Key Laboratory of Research on Emergency in TCM, Guangzhou, 510120 China

**Correction to: Trials 22, 188 (2021)**

**https://doi.org/10.1186/s13063-021-05113-y**

Following publication of the original article [1], we have been notified of a correction to Fig. 1 (Excluded non-TCM trials COVID-19).

• Originally published Fig. 1:

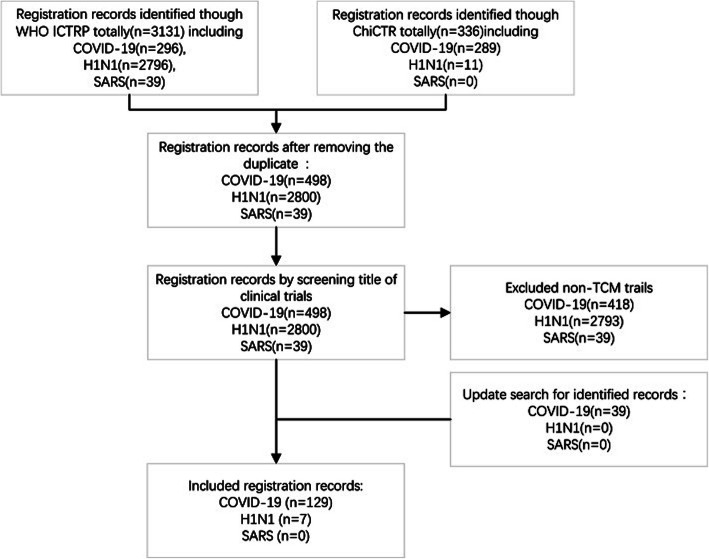



• Corrected Fig. 1:

**Fig. 1:**
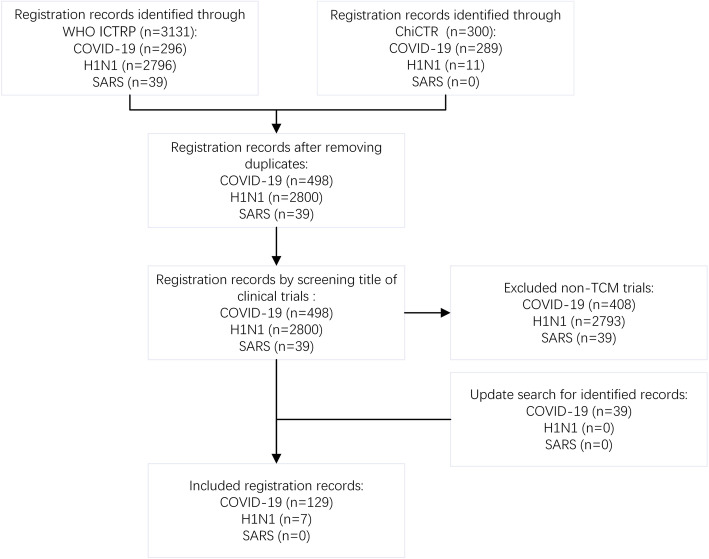
Flow diagram of record screening. ICTRP, International Clinical Trials Registry Platform; ChiCTR, Chinese Clinical Trial Registry; TCM, traditional Chinese medicine; COVID-19, coronavirus disease 2019; H1N1, H1N1 influenza; SARS, severe acute respiratory syndrome

The original article has been corrected.
