# Supplementary material for: Calling for improved quality in the registration of traditional Chinese medicine during the public health emergency: a survey of trial registries for COVID-19, H1N1, and SARS
Source: Trials. 2021 Mar 5;22:188. doi: 10.1186/s13063-021-05113-y (PMC7934977; doi:10.1186/s13063-021-05113-y)
Supplement: Supplementary file 1 — Additional file 1. Search strategy. [file 13063_2021_5113_MOESM1_ESM.docx]

**Search strategy of WHO ICTRP：**

COVID-19: Novel coronavirus OR 2019-novel coronavirus OR Novel CoV OR 2019-nCoV OR 2019-CoV OR Wuhan seafood market pneumonia virus OR COVID-19 OR NCP

H1N1：H1N1 OR Influenza A Virus, H1N1 Subtype OR Influenza A OR Influenza virus A

SARS：Severe Acute Respiratory Syndrome OR SARS OR SARS-CoV OR SARS-Related OR SARS-Associated

**Search strategy of ChiCTR:**

COVID-19：COVID-19 /NCP/新型冠状病毒肺炎

H1N1：H1N1/ 甲流 /甲型流感/ 甲型H1N1流感

SARS：SARS/非典型肺炎/传染性非典型肺炎/严重急性呼吸综合征
