# Supplementary material for: Calling for improved quality in the registration of traditional Chinese medicine during the public health emergency: a survey of trial registries for COVID-19, H1N1, and SARS
Source: Trials. 2021 Mar 5;22:188. doi: 10.1186/s13063-021-05113-y (PMC7934977; doi:10.1186/s13063-021-05113-y)
Supplement: Supplementary file 2 — Additional file 2. Registration quality assessment of TCM CTs by using TRDS-TCM. [file 13063_2021_5113_MOESM2_ESM.docx]

**Supplementary material 2**

**Table. Registration quality assessment of TCM CTs by using TRDS-TCM (n, %)**

| Item name | COVID-19(n=129) | H1N1(n=7) | Total(n=136) |
| --- | --- | --- | --- |
| 1.Primary Registry and Trial  Identifying Number | 129(100.0%) | 7 (100.0%) | 136(100.0%) |
| 2.Date of Registration in Primary Registry | 129 (100.0%) | 7 (100.0%) | 136(100.0%) |
| 3.Secondary Identifying Numbers | 97 (75.2%) | 6 (85.7%) | 103(75.7%) |
| 4.Statement of whether any conflicts of interest exist | 127 (98.4%) | 7 (100.0%) | 134(98.5%) |
| 4.1 Statement of whether any conflicts of interest | 0(0%) | 0(0%) | 0(0%) |
| 5. Primary Sponsor | 129 (100.0%) | 7 (100.0%) | 136(100.0%) |
| 6. Secondary Sponsor(s) | 121 (93.8%) | 5 (71.4%) | 126(92.6%) |
| 7. Contact for Public Queries | 128 (99.2%) | 7 (100.0%) | 135(99.3%) |
| 8. Contact for Scientific Queries | 128 (99.2%) | 7 (100.0%) | 135(99.3%) |
| 9. Public Title | 129 (100.0%) | 7 (100.0%) | 136(100.0%) |
| 10. Scientific Title | 129 (100.0%) | 7 (100.0%) | 136(100.0%) |
| 10a. Statement of the trial targets a TCM Pattern，or a Western medicine–defined disease, or a Western medicine–defined disease with a specific TCM Pattern | 129(100%) | 7(100%) | 136(100%) |
| 10b. Illustration of the name of the TCM intervention | 66(51.1%) | 5(71.4%) | 71(52.2%) |
| 11. Countries of Recruitment | 129 (100.0%) | 7 (100.0%) | 136(100.0%) |
| 11.1 The research setting(s) or centre(s) from which participants will be, are being, or have been recruited at the time of registration. | 129(100%) | 6(85.7%) | 135(99.3%) |
| 12. Health Condition(s) or Problem(s) Studied | 129 (100.0%) | 7 (100.0%) | 136(100.0%) |
| 12.1 Including participants based on TCM pattern | 12(9.3%) | 2(28.6%) | 14(10.3%) |
| 13. Intervention(s) |  |  |  |
| 13.1Intervention name | 129 (100.0%) | 7 (100.0%) | 136(100.0%) |
| 13.2 Ingredients or detail technique | 13 (10.1%) | 1 (14.3%) | 14(10.3%) |
| 13.3 Form | 55 (42.6%) | 4 (57.1%) | 59(43.4%) |
| 13.4 Dosage and frequency | 20 (15.5%) | 4(57.1%) | 24(17.6%) |
| 13.5 Treatment duration | 6 (4.7%) | 0(0.0%) | 6(4.4%) |
| 13.6 Control group | 103 (79.8%) | 7(100.0%) | 110(80.9%) |
| Total average | 54.3 (42.1%) | 3.8(54.8%) | 58.1(42.7%) |
| 13a. Descriptions of TCM interventions | 10(7.8%) | 1(14.3%) | 11(8.1%) |
| 13b. Descriptions of control group(s) | 3(23.3%) | 0(0%) | 3(2.2%) |
| 13c. Statement of the qualifications or experiences criteria of possible treatment providers | 0(0%) | 0(0%) | 0(0%) |
| 14. Key Inclusion and Exclusion Criteria |  |  |  |
| 14.1 Criteria of inclusion and exclusion | 129 (100.0%) | 7(100.0%) | 136(100.0%) |
| 14.2 Gender | 129 (100.0%) | 7(100.0%) | 136(100.0%) |
| 14.3 Age | 109 (84.5%) | 7(100.0%) | 116(85.3%) |
| 14.4 Diagnosis criteria -Western medicine | 45 (34.9%) | 2(28.6%) | 47(34.6%) |
| 14.5 With healthy human volunteer | 1(0.8%) | 0(0.0%) | 1(0.7%) |
| Total average | 82.6(64.0%) | 4.6(65.7%) | 87.2(64.1%) |
| 14.6 Statement of whether participants with a specific TCM Pattern will be recruited | 0(0%) | 0(0%) | 0(0%) |
| 15. Study Type |  |  |  |
| 15.1Type of study | 129(100.0%) | 7(100.0%) | 136(100.0%) |
| 15.2 Method of allocation (randomized/non-randomized) | 107(82.9%) | 5(71.4%) | 112(82.4%) |
| 15.3 Masking (is masking used and, if so, who is masked) | 46(35.7%) | 2(28.6%) | 48(35.3%) |
| 15.4 Assignment (single arm, parallel, crossover or factorial) | 107(82.9%) | 7(100.0%) | 114(83.8%) |
| 15.5 Allocation concealment mechanism | 6(4.7%) | 0(0.0%) | 6(4.4%) |
| 15.6 Phase (if applicable) | 79(61.2%) | 5(71.4%) | 84(61.8%) |
| Total average | 79.0(61.2%) | 4.3(61.9%) | 83.3(61.3%) |
| 16. Date of First Enrollment | 129 (100.0%) | 7 (100.0%) | 136(100.0%) |
| 17. Sample Size | 129 (100.0%) | 7 (100.0%) | 136(100.0%) |
| 18. Recruitment Status | 129 (100.0%) | 7 (100.0%) | 136(100.0%) |
| 19. Primary Outcome(s) |  |  |  |
| 19.1Name | 128(99.2%) | 7(100.0%) | 135(99.3%) |
| 19.2Measurement of Primary Outcome(s) | 2(1.6%) | 0(0.0%) | 2(1.5%) |
| 19.3Timepoint of measurements | 17(13.2%) | 1(14.3%) | 18(13.2%) |
| Total average | 49.0(38.0%) | 2.7(38.1%) | 51.7(38.0%) |
| 20. Key Secondary Outcomes |  |  |  |
| 20.1Name | 89(69.0%) | 7(85.7%) | 96(70.6%) |
| 20.2Measurements of Key Secondary Outcomes | 2(1.6%) | 0(0.0%) | 2(1.5%) |
| 20.3Timepoint of measurements | 15(11.6%) | 1(14.3%) | 16(11.8%) |
| Total average | 35.3(27.4%) | 2.7(38.1%) | 38.0(27.9%) |
| 19-20. TCM-related outcome | 45(34.9%) | 2(28.6%) | 47(34.6%) |
| 21. Ethics Review |  |  |  |
| 21.1Ethics Review status | 125(96.9%) | 6(83.3%) | 131(96.3%) |
| 21.2 Date of approval | 98(76.0%) | 4(69.0%) | 102(75.0%) |
| 21.3 Name of Ethics committee(s) | 105(81.4%) | 4(78.6%) | 109(80.1%) |
| 21.4 Contact details of Ethics committee(s) | 104(80.6%) | 0(40.5%) | 104(76.4%) |
| Total average | 108.0 (83.7%) | 4.0(57.1%) | 112.0(82.4%) |
| 22. Completion date | 119 (92.2%) | 6 (85.7%) | 125(91.9%) |
| 23. Summary Results |  |  |  |
| 23.1 Date of posting of results summaries | 0(0%) | 0(0%) | 0(0%) |
| 23.2 Date of the first journal publication of results | 0(0%) | 0(0%) | 0(0%) |
| 23.3 URL hyperlink(s) related to results or a full reference list of publications | 0(0%) | 0(0%) | 0(0%) |
| 23.4 Baseline Characteristics | 0(0%) | 0(0%) | 0(0%) |
| 23.5 Participant flow | 0(0%) | 0(0%) | 0(0%) |
| 23.6 Adverse events | 0(0%) | 0(0%) | 0(0%) |
| 23.7 Outcome measures | 0(0%) | 0(0%) | 0(0%) |
| 23.8 URL link to protocol file(s) with version and date | 0(0%) | 0(0%) | 0(0%) |
| 23.9 Brief summary | 0(0%) | 0(0%) | 0(0%) |
| Total average | 0(0%) | 0(0%) | 0(0%) |
| 24. Data sharing plan | 122(94.6%) | 6 (85.7%) | 128(94.1%) |
| A1. URL | 0(0%) | 0(0%) | 0(0%) |
| B1. Lay Summary  TCM specific background, rationale, theoretical origin | 0(0%) | 0(0%) | 0(0%) |
| C1. Approvals | 102(79.1%) | 4(57.1%) | 107(78.7%) |
| Note: COVID-19, severe acute respiratory syndrome coronavirus 2; H1N1, H1N1 influenza; TCM, traditional Chinese medicine. TRDS-TCM: WHO Trial Registration Data Set (TRDS) extension for traditional Chinese medicine 2020. | | | |
